# Supplementary material for: End Sequence Analysis Toolkit (ESAT) expands the extractable information from single-cell RNA-seq data
Source: Genome Res. 2016 Oct;26(10):1397–410. doi: 10.1101/gr.207902.116 (PMC5052061; doi:10.1101/gr.207902.116)
Supplement: Supplemental Material [file supp_gr.207902.116_Supp_Materials.tar.gz › ESAT-esat_v0.1/doc/html/functions_func_d.html]

ESAT: Class Members - Functions


ESAT


- Main Page
- Related Pages
- Packages
- Classes
- Files

- Class List
- Class Index
- Class Hierarchy
- Class Members

- All
- Functions
- Variables
- Enumerator

- a
- b
- c
- d
- e
- f
- g
- h
- i
- j
- k
- l
- m
- n
- o
- p
- q
- r
- s
- t
- u
- v
- w
- z


All Classes Namespaces Files Functions Variables Enumerator Pages

### - d -

- dataInverse()
  : broad.core.datastructures.MatrixWithHeaders
- decode()
  : broad.core.sequence.Sequence
- decodeShort()
  : broad.core.multiplealignment.MultipleAlignment
- DecollapseByIntronLocation()
  : broad.core.util.CollapseByIntersection
- DecollapseByIntronLocationOld()
  : broad.core.util.CollapseByIntersection
- DecollapseByIntronLocationWorking()
  : broad.core.util.CollapseByIntersection
- dedup()
  : broad.pda.annotation.BEDFileParser
  , broad.pda.gene.GeneWithIsoforms
- DefineGeneSignatures()
  : broad.projection.gsa.DefineGeneSignatures
- delete()
  : broad.pda.feature.genome.Chromosome
  , broad.pda.feature.genome.DirectoryInstalledGenomeAssembly
- destroyFileHandle()
  : broad.core.multiplealignment.MAFIO
- det()
  : Jama.LUDecomposition
  , Jama.Matrix
- diff()
  : broad.core.math.Statistics
- differenceByGenomicRegion()
  : broad.pda.annotation.BEDFileParser
- DifferentialExpression()
  : broad.pda.differentialExpression.DifferentialExpression
- digest()
  : broad.core.primer3.RestrictionEnzyme
- DirectoryInstalledGenomeAssembly()
  : broad.pda.feature.genome.DirectoryInstalledGenomeAssembly
- disect()
  : broad.core.annotation.BasicGenomicAnnotation
  , broad.core.sequence.SequenceRegion
  , umms.core.annotation.Annotation
  , umms.core.annotation.BasicAnnotation
- dispose()
  : broad.core.datastructures.JCSCache
- distance()
  : broad.pda.geneexpression.clustering.Kmeans
- distanceBetween()
  : broad.pda.datastructures.Alignments
- distanceFrom()
  : broad.core.motif.PositionWeightMatrix
- distanceToNearestNonOverlapper()
  : umms.core.annotation.Gene
- distanceToOppositeStrand5primeNeighbor()
  : broad.pda.annotation.Locus
- doesOverlap()
  : broad.core.datastructures.IntervalTree< V >
- doRepeatListNeedsCleanup()
  : broad.pda.feature.genome.DirectoryInstalledGenomeAssembly
- doWork()
  : broad.pda.samtools.FilterFastqReads
  , broad.pda.samtools.FilterPairedReadsByMAQ
  , broad.pda.samtools.PairedEndTDF
  , broad.pda.samtools.TrimFastqReads
  , broad.pda.seq.rap.BuildRatioNullDistribution
  , broad.pda.seq.rap.CalculateCoverageStats
  , broad.pda.seq.rap.CollectAnnotationEnrichments
  , broad.pda.seq.rap.CountProbes
  , broad.pda.seq.rap.CountReads
  , broad.pda.seq.rap.DistanceToBED
  , broad.pda.seq.rap.GCContent
  , broad.pda.seq.rap.PlotAggregateRegions
  , broad.pda.seq.rap.RatioPermutationPeakCaller
  , broad.pda.seq.rap.rna.CountReads
  , broad.pda.seq.rap.ShuffleBAM
  , broad.pda.seq.rap.ShuffleBED
  , broad.pda.seq.rap.SkellamKLPeakCaller
  , broad.pda.seq.rap.SlideAndCalculate
  , broad.pda.seq.rap.SlideAndCount
  , broad.pda.seq.rap.UnmarkDuplicates
  , umms.core.scripture.statistics.ConnectDisconnectedTranscripts
- drawAutosome()
  : broad.pda.feature.genome.DirectoryInstalledGenomeAssembly
- drawChromosome()
  : broad.pda.feature.genome.DirectoryInstalledGenomeAssembly
- drawInitialState()
  : broad.core.hmm.MarkovModel< T >
- drawNextState()
  : broad.core.hmm.MarkovModel< T >
- drawRandomAutosomeRegion()
  : broad.pda.feature.genome.DirectoryInstalledGenomeAssembly
- drawRandomRegion()
  : broad.pda.feature.genome.Chromosome
  , broad.pda.feature.genome.DirectoryInstalledGenomeAssembly


---

Generated on Fri Aug 15 2014 15:11:16 for ESAT by  

 1.8.7
